# Caspase/AIF/apoptosis pathway: a new target of puerarin for diabetes mellitus therapy

**DOI:** 10.1007/s11033-019-04925-1

**Published:** 2019-06-21

**Authors:** Tao Liang, Xiaohui Xu, Dongmei Ye, Wenxia Chen, Biyun Gao, Yanjun Huang

**Affiliations:** 1 College of Stomatology of Guangxi Medical University, No. 10, Shuangyong Road, Nanning, 530021 People’s Republic of China; 2 Affiliated Tumor Hospital of Guangxi Medical University, Institute of Cancer Prevention and Treatment of Guangxi Zhuang Autonomous Region, Nanning, 530021 Guangxi People’s Republic of China; 3 Department of Clinical Pharmacy, Hospital of Guangxi Zhuang Autonomous Region, Nanning, 530021 Guangxi People’s Republic of China; 4 Guangxi Medical University, Nanning, 530021 Guangxi People’s Republic of China

**Keywords:** Puerarin, Type 2 diabetes mellitus, Apoptosis-inducing factor, Caspase

## Abstract

Pancreatic β cell damage is one of the crucial factors responsible for the development of type 2 diabetes mellitus (T2DM). Previous studies have suggested that puerarin (PR) could regulate the activities of the mitochondrial respiratory chain complex in diabetic nephropathy (DN); however, whether PR can inhibit pancreatic β-cell apoptosis in T2DM remains to be elucidated. In the present study, T2DM mice induced by high-fat diet and streptozotocin (STZ) injection were used as a working model to investigate the mechanism of PR on pancreatic β cell apoptosis. The results showed that PR decreased the serum fasting blood glucose (FBG), total cholesterol (TC), triglyceride (TG) and low-density lipoprotein (LDL) levels but significantly increased the fasting blood insulin (FINS) and high-density lipoprotein (HDL) levels. Furthermore, decreased caspase-3, 8, 9 and apoptosis-inducing factor (AIF) proteins in the pancreas were detected by Western blot analysis. Terminal deoxynucleotidyl transferase-mediated dUTP nick end labelling (TUNEL) staining demonstrated that the pancreatic β cell apoptosis was inhibited by PR. Furthermore, PR improved the histopathological changes in pancreatic tissue in T2DM mice. Collectively, the data show that PR can protect the β cells from apoptotic death in a mouse model of T2DM through regulating the expression of apoptosis-related protein-AIF and caspase family proteins.

## Introduction

T2DM is an endocrine disease that often accompanies other metabolic disorders, which are potentially life threatening [1]. Insulin resistance (IR) occurs in the early stage of T2DM and gradually leads to diminished insulin secretory ability of β cells due to structural damage that finally results in glucose and lipid metabolism disorders [2]. As is known, mitochondrial oxidative stress is a key factor contributing to IR and β cell dysfunction. Excess reactive oxygen species (ROS) could activate down-stream apoptotic factors including cytochrome C (Cyto-C) and AIF and induce β cell apoptosis [3, 4]. In our previous studies, we discovered that PR could decrease the FBG level of STZ-induced diabetic mice through ameliorating oxidative stress and reducing the expression of inflammatory factors nuclear-factor kappa B (NF-κB) and Cyto-C [5]. We therefore set out to explore the mechanism of PR on pancreas apoptosis in T2DM mice.

Traditionally, apoptosis is executed by the caspase protease family of proteins, which are activated through the exogenous death receptor pathway and endogenous mitochondrial pathway. The caspase family proteins increase the mitochondrial permeability, which then triggers Cyto-C release from the mitochondria and the formation of an apoptosis-inducing complex with Apaf-1, ATP and pro-caspase-9 [6, 7]. Previous studies have found that mitochondrial apoptosis-inducing factor (AIF) can mediate nuclear apoptosis [8, 9]. AIF is thought to play a central role in the caspase-independent apoptosis pathway. The pro-apoptotic effect of AIF is also reflected in its own positive feedback, that is, AIF released into the cytoplasm can act on other mitochondria, increasing their permeability and further promoting the release of AIF. AIF also promotes the release of Cyto-C, ultimately activating caspase-3. In our preliminary study, we discovered that PR could induce the activation of Bcl-2, a regulatory factor of AIF, which indicates that PR may inhibit apoptosis by regulating the expression of AIF. Therefore, we aimed to test this hypothesis to further elucidate the mechanism of PR on T2DM.

STZ is a classic drug to induce diabetes in mice. Mice fed a high fat diet and administered STZ show clinical manifestations of hyperglycaemia, hyperlipidaemia, obesity and hyperinsulinaemia [10]. Due to its instability, the STZ solution must be freshly prepared and directly injected into overnight fasted mice via the tail vein.

PR, one of the active compounds of *Pueraria lobata,* has been promoted as a therapy for DM through its role in elevating insulin expression and maintaining metabolic homoeostasis in STZ-induced diabetic mice. In the present study, we explore the effect of PR on T2DM. We demonstrate for the first time that PR could inhibit pancreatic cell apoptosis in T2DM mice through regulating the expression of caspase family proteins and AIF.

## Materials and methods

### Animals

All the animal protocols were approved by the Institutional Ethics Committee of Guangxi Medical University (Approval No. 2012011121). All the animal experiments are conducted in accordance with the guidelines of the Guiding Opinions on The Treatment of Experimental Animals for the care and use of laboratory animals. Healthy male Kunming mice weighing approximately 18–22 g were purchased from the Experimental Animal Center of Guangxi Medical University (Registration No. SCXK 2010-0002). The animals were acclimatized under temperature-controlled (22–25 °C) laboratory conditions with a 12 h light–dark cycle and were given free access to tap water and standard rodent chow.

### Materials

**Fig. 1:**
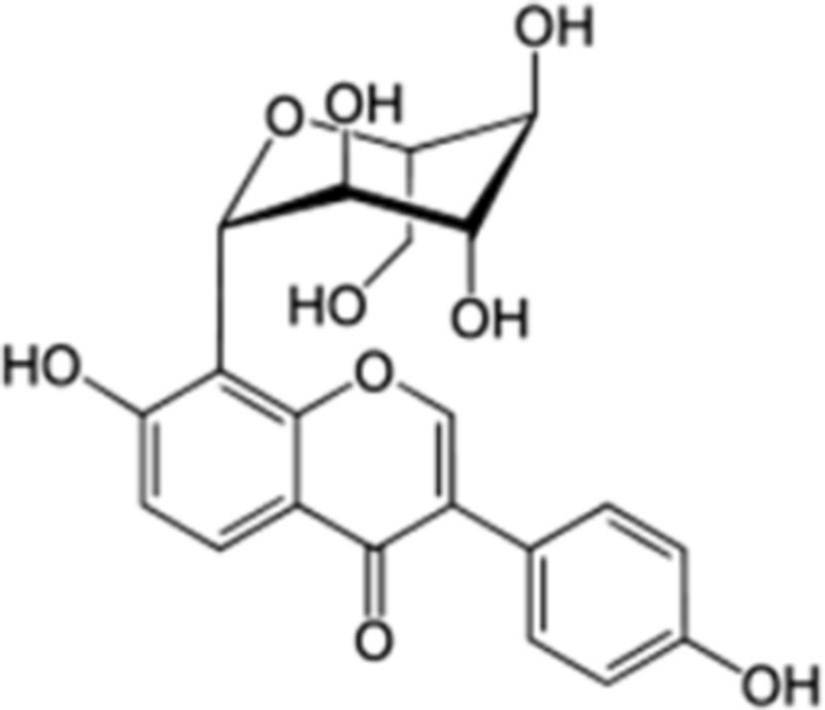
The chemical structure of puerarin isolated from *P. lobata* (Willd.)

A puerarin preparation (purity > 99%) was provided by the Department of Pharmaceutical Chemistry, Guangxi Medical University (Nanning, China). Metformin was purchased from Zhongxin Pharmaceutical Co., Ltd. (Tianjin, China). Streptozotocin (STZ) was obtained from Sigma Co., Ltd. (Missouri, USA). The molecular structure of PR is shown in Fig. 1. FBG and blood chemistry were measured with the Roche ACCU-CHEK^®^ Performa (Strip lot: 470664, Switzerland) and an automatic biochemical analyser (Hitachi Model 7100 Automatic Analyzer), respectively. The other materials are outlined in the following sections.

### Experimental design [11]

Type 2 diabetic mice were established by high-fat diet feeding and STZ injection. Healthy male Kunming mice were fed a high-fat diet for 1 month and were intravenously injected with 80 mg kg^−1^ body weight STZ after a 12 h fast. The freshly-prepared STZ solution was dissolved in refrigerated saline in light-free. And metformin is dissolved directly in saline for oral gavage. Seventy-two hours later, FBG was measured, and the mice with FBG ≥ 11.1 mmol L^−1^ were considered as T2DM mice. In addition, healthy male Kunming mice fed a standard rodent chow served as normal controls. The experimental animals were divided into the following groups:

- *Group 1* healthy mice treated with a saline solution by gavage: normal control.
- *Group 2* Type 2 diabetic mice treated with a saline solution by gavage: model control.
- *Group 3* Type 2 diabetic mice treated with 320 mg kg^−1^ metformin by gavage: positive control.
- *Group 4* Type 2 diabetic mice treated with 80 mg kg^−1^ PR by gavage (our previous study found that 80 mg kg^−1^ of PR is an effective dose [5]).

Metformin and PR were given daily at the same time for up to 15 days.

### Biochemical measurements

The FBG levels were detected during the experiment on day 0, 7 and 15 of treatment using the Roche ACCU-CHEK^®^ Performa through tail vein blood. The serum samples were collected from whole blood by centrifugation at 1300×*g* for 10 min for detection. The serum FINS content was measured by the Cusabio mouse ELISA kits (Huamei Biotech Co., Ltd., Hubei, China). The serum levels of TC, TG, LDL and HDL were analysed using commercially available kits (Jiancheng Bioengineering Institute, Nanjing, China).

### Pathological examination

An abdominal incision was performed to harvest the pancreas. Pancreatic tissues were fixed in 10% paraformaldehyde for 24 h and then embedded in paraffin. The sections (5 μm) were subjected to regular haematoxylin-eosin (HE) staining and observed under a light microscope Olympus CX4 (Japan).

### Transmission electron microscopy

The pancreas tissues used for electron microscopy examination were removed under 0 °C, were cut into small pieces and immediately fixed in 2.5% pre-cooled glutaraldehyde. Ultrathin sections (70 nm) were subjected to uranylacetate and lead citrate staining. Finally, the samples were observed under a transmission electron microscopy (Hitachi H-7650).

### Pancreatic β cell apoptosis assay

The in situ cell death detection kit (Roche, Germany) was applied to the pancreas tissue for terminal deoxynucleotidyl transferase-mediated dUTP nick end labelling (TUNEL) staining. The apoptotic cells were detected under the observation of a light microscope through the colour reaction.

### Western blot analysis

The pancreas samples were homogenized in lysis buffer and total protein was extracted. The protein concentrations were determined using a protein assay reagent (Bio-Rad). For Western blot analysis, the protein lysates were resolved by SDS–polyacrylamide gel electrophoresis and transferred onto polyvinyldifluoride membranes. The membranes were blocked with PBST buffer (1% Tween-20, PBS) for 2 h at room temperature and then incubated with primary antibodies against caspase-3, 8, 9 and AIF (1:1000 Santa Cruz, USA) overnight at 4 °C. After three washes, the blots were incubated with a goat anti-rabbit and/or goat anti-mouse horseradish peroxidase-conjugated secondary IgG (Boster Biotechnology) for 2 h at room temperature. The immunoreactive bands were visualized with diaminobenzidine. The representative bands were measured by Scion image software (Scion Corp., Frederick, MD). The protein levels were normalized to those of β-actin.

### Statistical analyses

The data are expressed as the mean ±  S.E. The significant differences between the groups were analysed with one-way ANOVAs followed by Tukey’s tests for comparisons between groups using SPSS16.0. *P*-values < 0.05 were considered statistically significant.

## Results

### Effect of PR on serum levels of FBG and insulin in T2DM mice

**Fig. 2:**
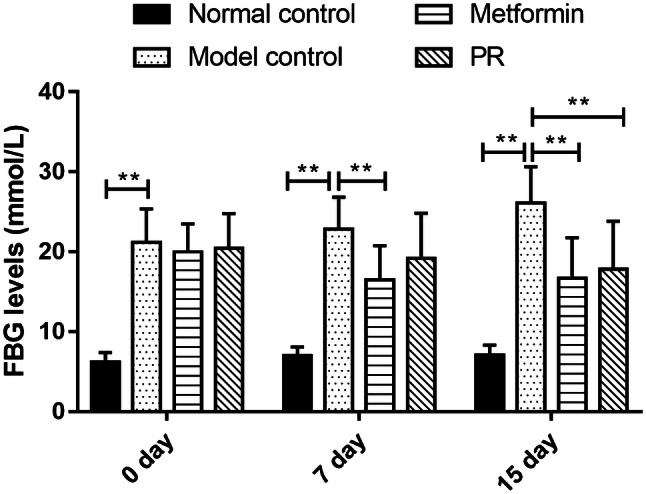
Hypoglycaemic effect of PR. The results are presented as the mean ± S.E (n = 10). *P < 0.05 compared with the model group; **P < 0.01 compared with the model group

**Fig. 3:**
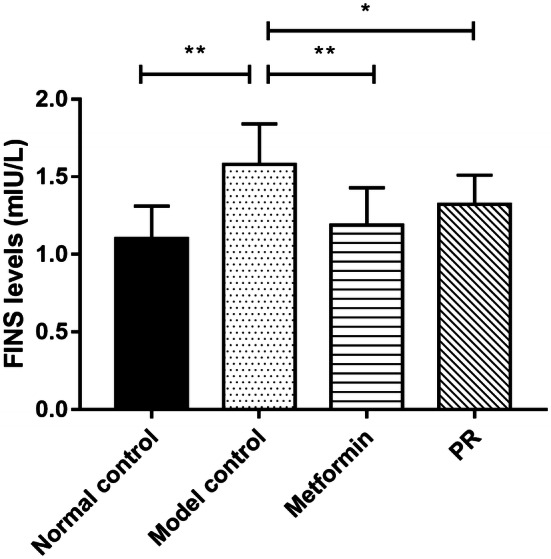
Effects of PR on FINS. The serum samples were collected on the 15th day. The results are presented as the mean ± S.E (n = 10). *P < 0.05 compared with the model group; **P < 0.01 compared with the model group

To investigate the hypoglycaemic effect of PR on T2DM mice, we administered 80 mg kg^−1^ PR orally for 15 days to high fat diet-fed and STZ-induced T2DM Kunming mice. As shown in Fig. 2 and Table 1, the oral administration of PR effectively decreased the FBG level relative to T2DM mice compared with the model control group, as well as the metformin-treated group. The FINS content significantly decreased in the T2DM mice administered metformin compared with the model controls. FINS were decreased in mice treated with PR showing difference (Fig. 3 and Table 1).

**Table 1:** Effect of PR on serum levels of FBG and insulin in T2DM mice

Groups	FBG (mmol L^−1^)			FINS (mIU L^−1^)
	0 day	7 day	15 day	
Normal control	6.18 ± 1.21**	7.01 ± 1.06**	7.08 ± 1.24**	1.1 ± 0.21**
Model control	21.15 ± 4.21	22.8 ± 4.02	26.1 ± 4.50	1.58 ± 0.26
Metformin	19.97 ± 3.52	16.49 ± 4.26**	16.66 ± 5.08**	1.19 ± 0.24**
PR	20.44 ± 4.33	19.15 ± 5.67	17.82 ± 6.01**	1.32 ± 0.19*

Data are expressed as the mean ± S.E. (n = 10)

*P < 0.05 compared with the model group

**P < 0.01 compared with the model group

### Effect of PR on serum lipid profiles in T2DM mice

**Fig. 4:**
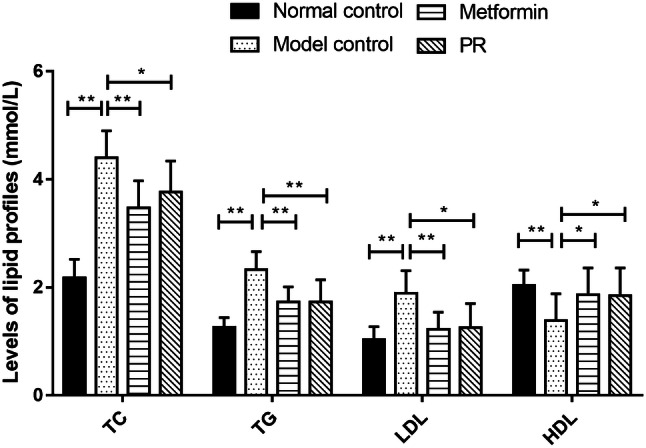
Effects of PR on lipid profiles. The serum samples were collected on the 15th day. The results are presented as the mean ± S.E (n = 10). *P < 0.05 compared with the model group; **P < 0.01 compared with the model group

Hyperglycaemia is often accompanied by hyperlipidaemia. Thus, we also measured the TC, TG, LDL and HDL levels to observe changes in lipid metabolism in T2DM mice. The serum levels of TC, TG and LDL were reduced by PR treatment compared to untreated diabetic mice. In addition, PR treatment enhanced the HDL level in diabetic mice compared with model controls (Fig. 4 and Table 2).

**Table 2:** Effect of PR on serum lipid profiles in T2DM mice

Lipid profiles (mmol/L)	Normal control	Model control	Metformin	PR
TC	2.18 ± 0.34**	4.4 ± 0.50	3.47 ± 0.51**	3.76 ± 0.58*
TG	1.26 ± 0.18**	2.33 ± 0.33	1.73 ± 0.28**	1.73 ± 0.41**
LDL	1.03 ± 0.24**	1.89 ± 0.42	1.22 ± 0.32**	1.25 ± 0.45*
HDL	2.04 ± 0.28**	1.38 ± 0.50	1.86 ± 0.50*	1.84 ± 0.52*

Data are expressed as the mean ± S.E. (n = 10)

*P < 0.05 compared with the model group

**P < 0.01 compared with the model group

### Effect of PR on morphological changes in the pancreas of T2DM mice

**Fig. 5:**
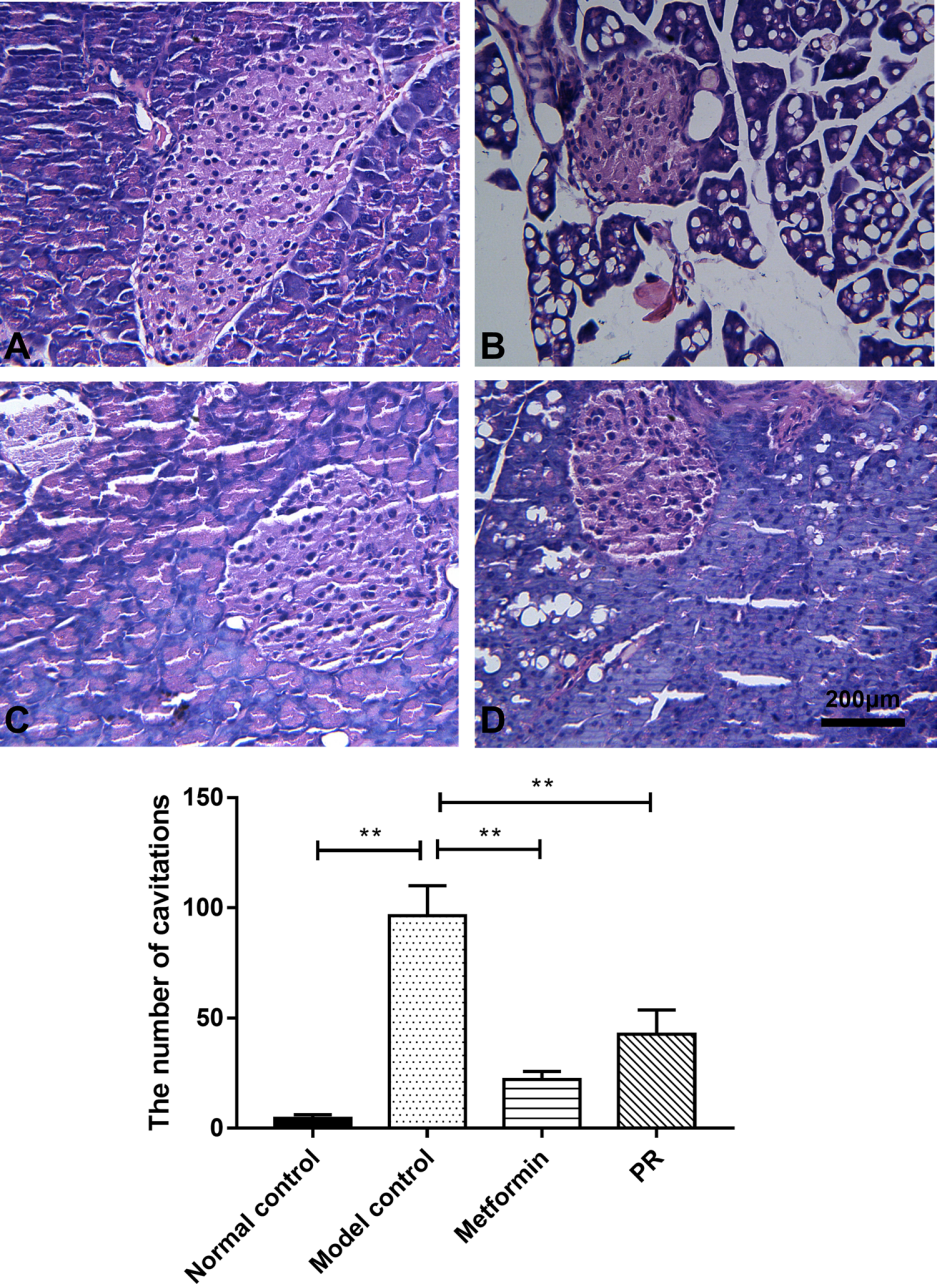
Pathophysiological observation of the pancreas (HE stain, n = 10, × 400). The pancreas samples were collected on the 15th day. **a** Normal control; **b** model control; **c** 320 mg kg^−1^ d^−1^ of metformin; **d** 80 mg kg^−1^ d^−1^ of PR. The results are presented as the mean ± S.E. *P < 0.05 compared with the model group; **P < 0.01 compared with the model group

HE-stained pancreatic tissue showed that the cellular structure was maintained in healthy mice. Compared with the islets in normal samples, sparseness and cavitations were observed in the model control group due to the damage caused by STZ injection. The damage was alleviated by the administration of PR as shown in Fig. 5 and Table 3.

**Table 3:** The number of cavitations in HE results of pancreas

Groups	Number of cavitations
Normal control	4.56 ± 1.62**
Model control	96.43 ± 13.58
Metformin	22.14 ± 3.64**
PR	42.68 ± 11.05**

Data are expressed as the mean ± S.E. (n = 10)

*P < 0.05 compared with the model group

**P < 0.01 compared with the model group

### Effect of PR on the pancreatic ultrastructure in T2DM mice

**Fig. 6:**
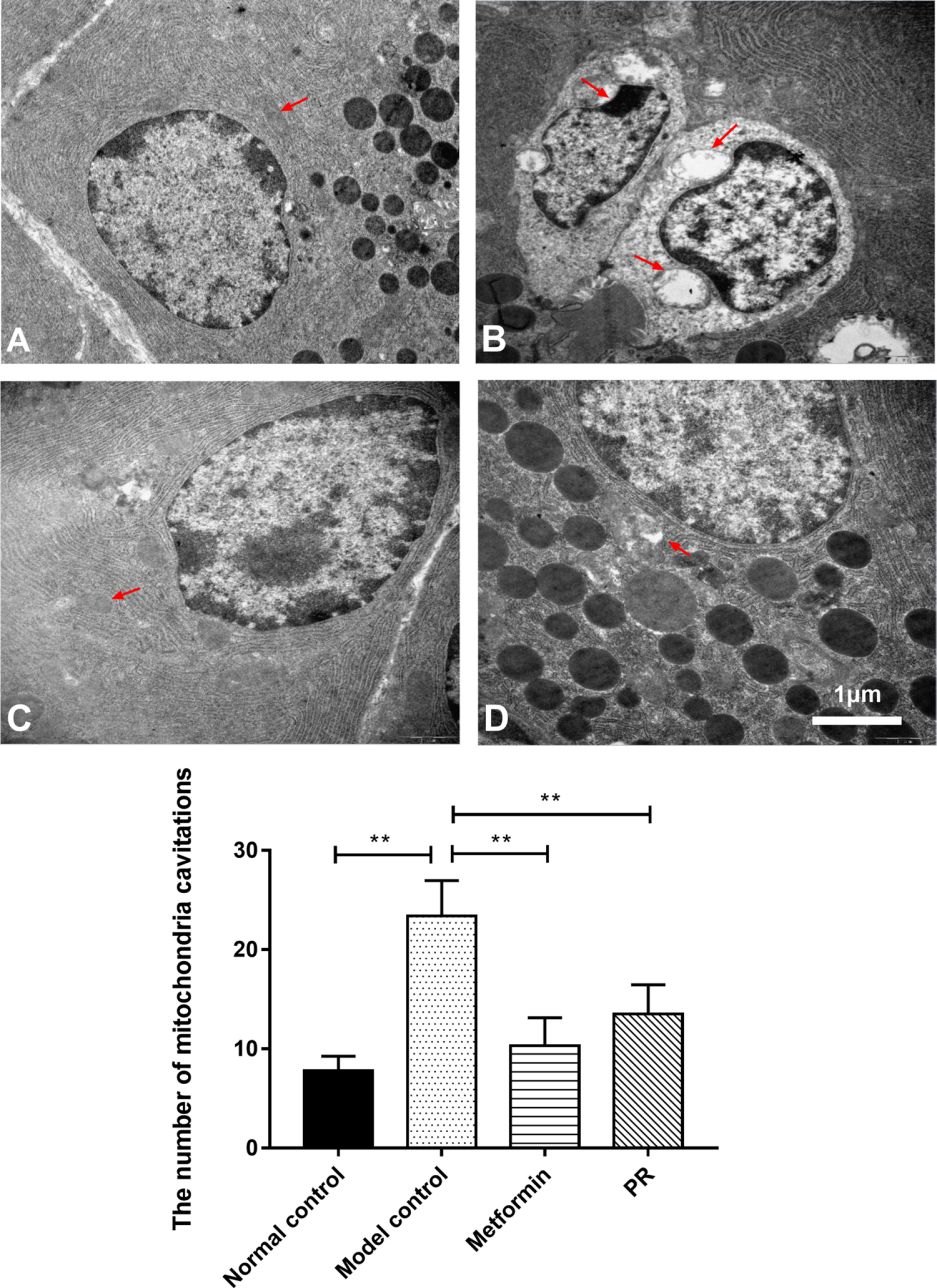
Observation of pancreatic ultrastructure. The pancreas samples were collected on the 15th day. The red arrows point to the mitochondria in the pancreatic cells. **a** Normal control; **b** model control; **c** 320 mg kg^−1^ d^−1^ of metformin; **d** 80 mg kg^−1^ d^−1^ of PR. The results are presented as the mean ± S.E (n = 10). *P < 0.05 compared with the model group; **P < 0.01 compared with the model group

Ultrastructure observation provides a clear and direct view of organelles. As shown in Fig. 6 and Table 4, nuclear pyknosis and deformation features of apoptosis, as well as mitochondrial cavitation, were observed in the islet cells of diabetic mice. By contrast, T2DM mice treated with PR exhibited relatively fewer nuclear deformations and cavitated mitochondria.

**Table 4:** The number of mitochondria cavitations in ultrastructure observation of pancreas

Groups	Number of mitochondria cavitations
Normal control	7.8 ± 1.45**
Model control	23.4 ± 3.57
Metformin	10.3 ± 2.84**
PR	13.5 ± 2.97**

Data are expressed as the mean ± S.E. (n = 10)

*P < 0.05 compared with the model group

**P < 0.01 compared with the model group

### Effect of PR on pancreatic apoptosis in T2DM mice

**Fig. 7:**
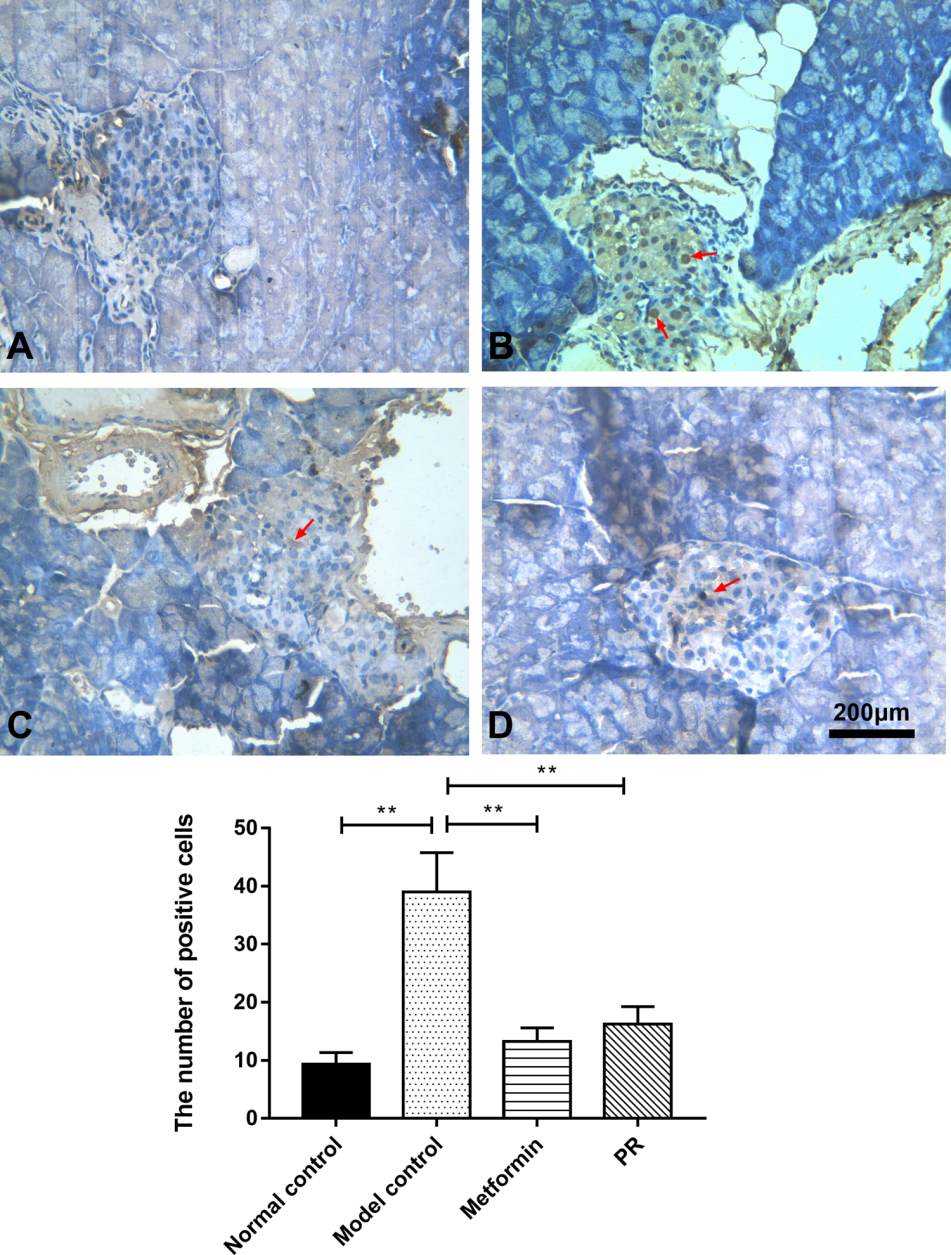
Observation of pancreatic apoptosis. The pancreas samples were collected on the 15th day. The red arrows point to the TUNEL-positive cells. **a** Normal control; **b** model control; **c** 320 mg kg^−1^ d^−1^ of metformin; **d** 80 mg kg^−1^ d^−1^ of PR. The results are presented as the mean ± S.E (n = 10). *P < 0.05 compared with the model group; **P < 0.01 compared with the model group

TUNEL staining, in which positive cells appear brown, was conducted to investigate apoptosis in the pancreas. As shown in Fig. 7 and Table 5, the number of positive cells was greater in the T2DM mice compared to the normal controls. Treatment with PR as well as metformin significantly attenuated the decreased cell viability.

**Table 5:** The number of positive cells in TUNEL staining of pancreas

Groups	Number of positive cells
Normal control	9.34 ± 2.03**
Model control	39.01 ± 6.78
Metformin	13.26 ± 2.33**
PR	16.23 ± 3.05**

Data are expressed as the mean ± S.E. (n = 10)

*P < 0.05 compared with the model group

**P < 0.01 compared with the model group

### Effect of PR on the protein expressions of caspase-3, 8, 9 and AIF in T2DM mice

**Fig. 8:**
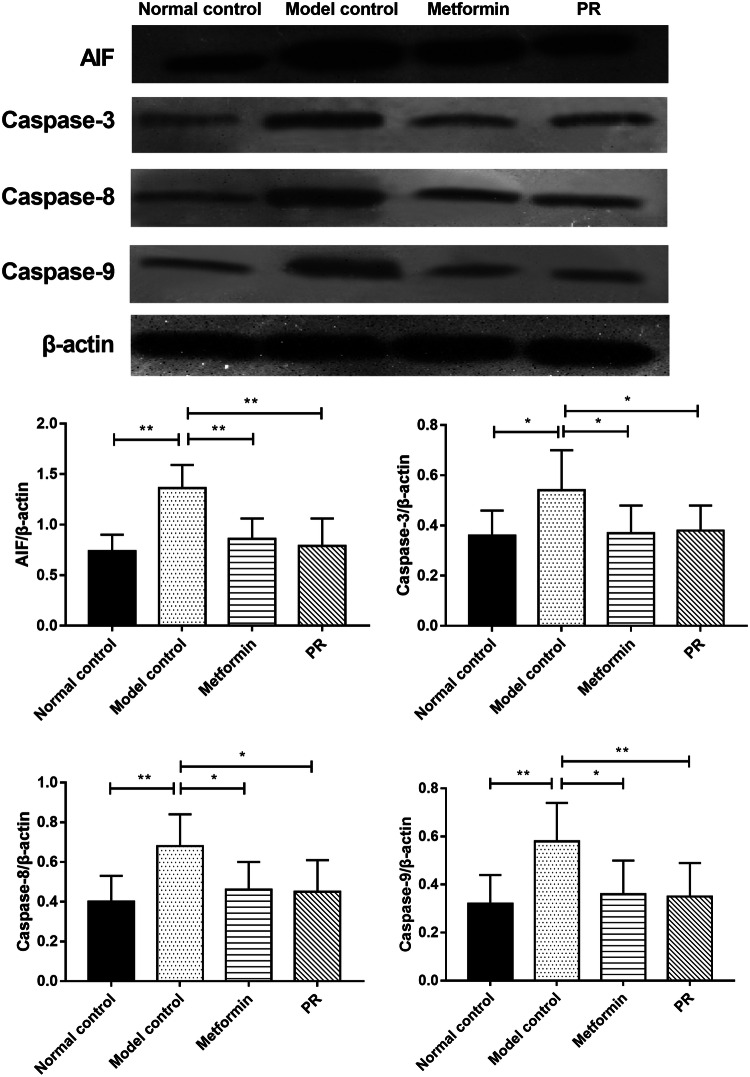
Effects of PR on the protein expression of caspase-3, 8, 9 and AIF. The pancreas samples were collected on the 15th day. The results are presented as the mean ± S.E (n = 10). *P < 0.05 compared with the model group; **P < 0.01 compared with the model group

The caspase family of proteins is involved in inducing apoptosis. AIF is also a crucial factor responsible for mitochondrial apoptosis. Hence, we investigated whether PR affected the expression of caspase family proteins and AIF in T2DM mice. As shown in Fig. 8 and Table 6, increased caspase-3, 8, 9 and AIF in the pancreatic tissues from diabetic mice was observed. PR treatment effectively abolished the elevated protein expression levels of the caspase family proteins and AIF.

**Table 6:** The protein expressions of caspase-3, 8, 9 and AIF in T2DM mice

Target gene/β-actin	Normal control	Model control	Metformin	PR
AIF/β-actin	0.74 ± 0.16**	1.36 ± 0.23	0.86 ± 0.20**	0.79 ± 0.27**
Caspase-3/β-actin	0.36 ± 0.10*	0.54 ± 0.15	0.37 ± 0.11*	0.38 ± 0.10*
Caspase-8/β-actin	0.40 ± 0.13**	0.68 ± 0.16	0.46 ± 0.14*	0.45 ± 0.16*
Caspase-9/β-actin	0.32 ± 0.12**	0.58 ± 0.17	0.36 ± 0.13*	0.35 ± 0.10**

Data are expressed as the mean ± S.E. (n = 10)

*P < 0.05 compared with the model group

**P < 0.01 compared with the model group

## Discussion

Relative insulin deficiency is a well-known crucial factor in the progression of T2DM that contributes to the damage and apoptosis of islet β cells [12]. Thus, islet β cell repair is an efficient therapeutic target for T2DM treatment. In the present study, we demonstrated that PR can attenuate STZ-induced pancreatic cell apoptosis by inhibiting the expression of caspase family proteins and AIF.

PR, an isoflavonoid, is the main active component of *P. lobata.* PR has been reported to possess anti-oxidative, hypotensive and hypoglycaemic activities [13–15]. Prior studies have demonstrated that PR exerted protective effects on diabetic kidney injury, heart failure, diabetic cardiomyopathy, cardiac fibroblast proliferation, skeletal muscle energy metabolism and liver lipid metabolism [16–22]. The hypoglycaemic effect of PR on diabetes mellitus was confirmed by previous studies [15, 23, 24]. In our former study, PR treatment decreased the FBG level in diabetic nephropathy mice by attenuating oxidative stress. Another prior study found that PR is distributed in the pancreas, which may explain the hypoglycaemic manifestation of PR in diabetes [25].

High glucose-induced oxidative stress in diabetes would affect the regulation of insulin, which may lead to a reduction in insulin synthesis and promote β cell dysfunction. Mitochondria are damaged when exposed to excessive oxygen free radicals, which disturb the respiratory energy pathways [26]. Blood glucose control is the basis of DM treatment. In addition, β cell damage could be a therapeutic target. To detect the effect of PR on the pancreas, we examined the pancreatic ultrastructure and measured the expression of apoptosis-related proteins.

Previous research discovered two pathways that link mitochondrial and nuclear apoptosis: first, the AIF-dependent pathway, in which a large fragment of DNA breaks and peripheral chromosome agglutination occur upon AIF activation; second, the caspase-dependent pathway, where the production of oligonucleotide-like DNA fragments and further chromosome agglutination rely on the activation of Cyt-C, Apaf-1, caspase and CAD [26–28]. Caspase-3 is considered the most important protein in the caspase-dependent apoptosis pathway because it plays a key role in the initiation of apoptosis. Moreover, AIF is also thought to play a central role in the regulation of caspase-independent apoptosis. However, the investigation by Arnoult D reported that the apoptotic effect of AIF is dependent on caspase; study results showed that a caspase inhibitor had no effect on mitochondrial Cyt-C release, but AIF release was inhibited, and AIF exerted an apoptotic effect after the activation of caspase [29, 30]. Our WB results demonstrated that the expression of caspase-3, 8, 9 and AIF was reduced by PR, suggesting that PR prevents pancreatic cell apoptosis through inhibiting the caspase-dependent apoptotic pathway and AIF. These results corroborate our previous study showing that PR inhibited the expression of Cyt-C [5]. Together these data suggest that PR may prevent apoptosis by reducing the release of Cyt-C through regulating the caspase family proteins. Previous studies found that puerarin could inhibit the expression of caspase 3 in diabetic osteoblasts and glomerular epithelial cells, improving osteoporosis and glomerular fibrosis. Indicating that puerarin could inhibit apoptosis through regulating the caspase family [31, 32]. The results of present study are consistent with it; however, whether PR can inhibit both the caspase-dependent and caspase-independent AIF pathways remains to be further investigated.

The protective effect of PR on apoptosis was further confirmed by pathological and ultrastructure observations. PR treatment attenuated abnormal islet morphology including the shape and integrity of the cells. Specifically, nuclear deformation and mitochondrial cavitation were reduced following PR treatment. These effects may be due to the inhibition of caspase-3, 8, 9 and the AIF pathway; however, the AIF downstream signalling pathway still needs further verification.

Collectively, our findings provide new insights into the pathogenic process of pancreas injury and identify PR as a new therapy targeting the caspase/AIF/apoptosis pathway.

## Abbreviations

T2DM: Type 2 diabetes mellitus
PR: Puerarin
STZ: Streptozotocin
FBG: Fasting blood glucose
TC: Total cholesterol
TG: Triglyceride
LDL: Low-density lipoprotein
FINS: Fasting blood insulin
HDL: High-density lipoprotein
AIF: Apoptosis-inducing factor
TUNEL: Terminal deoxynucleotidyl transferase-mediated dUTP nick end labelling
IR: Insulin resistance
ROS: Reactive oxygen species
Cyto-C: Cytochrome C
NF-κB: Nuclear-factor kappa B
HE: Haematoxylin–eosin
